# FEM simulations for double diffusive transport mechanism hybrid nano fluid flow in corrugated enclosure by installing uniformly heated and concentrated cylinder

**DOI:** 10.1038/s41598-023-50659-3

**Published:** 2024-01-08

**Authors:** S. Bilal, Imtiaz Ali Shah, Ilyas Khan, Shaha Al-Otaibi, Ariana Abdul Rahimzai

**Affiliations:** 1 School of Mathematics and Statistics, Central South University, Changsha, 410083 People’s Republic of China; 2 Department of Mathematics, Air University, P.A.F Complex E-9, Islamabad, 44000 Pakistan; 3 Department of Mathematics, College of Science, Al-Zulfi, Majmaah University, 11952 Al-Majmaah, Saudi Arabia; 4 Department of Information Systems, College of Computer and Information Sciences, Princess Nourah Bint Abdulrahman University, P. O. Box 84428, 11671 Riyadh, Saudi Arabia; 5 Department of Mathematics, Education Faculty, Laghman University, Mehtarlam, 2701 Laghman Afghanistan

**Keywords:** Mathematics and computing, Nanoscience and technology

## Abstract

Generation of fluid flow due to simultaneous occurrence of heat and mass diffusions caused by buoyancy differences is termed as double diffusion. Pervasive applications of such diffusion arise in numerous natural and scientific systems. This article investigates double diffusion in naturally convective flow of water-based fluid saturated in corrugated enclosure and containing hybrid nano particles composed of Copper (Cu) and Alumina (Al_2_O_3_). Impact of uniformly applied magnetic field is also accounted. To produce thermosolutal convective potential circular cylinder of constant radius is also adjusted by providing uniform temperature and concentration distributions. Finite element approach is capitalized to provide solution of utilized governing equations by utilizing Multiphysics COMSOL software. Wide-range of physical parameters are incorporated to depict their influence on associated distributions (velocity, temperature and concentration). Interesting physical quantities like Nusselt number, Sherwood numbers are also calculated against involved sundry parameters. It is note worthily observed that maximum strength of stream lines ${(\psi }_{max})$ is 3.3 at $\phi =0$ and drops to 1.2 when $\phi$ is increased to 0.04. Furthermore, in the hydrodynamic case (Ha = 0), it is observed that the velocity field exhibits an increasing trend compared to the hydromagnetic case $\left(Ha\ne 0\right),$ which is proved from the attained values of stream-function i.e., ${|\psi |}_{max}=11$ (in the absence of a magnetic field) and ${|\psi |}_{max}=3.5$ (in the presence of a magnetic field). It is revealed from the statistics of Nusselt number that increase in volume fraction of nano particles from 0 to 0.4, heat flux coefficient upsurges up to 7% approximately. Since, present work includes novel physical aspects of thermosolutal diffusion generated due to induction of hybrid nanoparticles in water contained in corrugated enclosure, so this study will provide innovative thought to the researchers to conduct research in this direction.

## Introduction

Diffusion in fluid flow phenomenon is generated due to thermosolutal gradients which are commonly caused by variation in buoyancy forces. In past years, researchers are of thought that control of heat transfer in domain is essential for efficient performance of multiple industrial procedures, but now a days with advancement in technologies strategies to control mass diffusion is also considered as a compulsion. Processes such as the drying process, filtration of solids from liquids, cooling of electronic equipment, HVAC systems, and heat exchangers are just a few examples of setups that can all benefit from thermosolutal diffusion. The gradients of both diffusion variables also arise frequently in many naturally existing processes like, the ice melting, intrusion of sea water in lakes, crystallization of magma intrusions in earth crust are popular examples. In addition, double diffusive convection is involved in production of natural gas and petroleum, grain and energy storages, moisture transport in large scale systems, cooling of molten metals, cleaning and dyeing processes and solidification of different equipment’s. Initiating from pioneering work performed by Sezai^1^, who described that consideration of both (heat and mass) diffusions is more realistic to existing physical systems because in the presence of density differences both gradients are generated in the flow simultaneously. Gebhart and Pera^2^ disclosed that thermal and mass gradients in laminar fluid flow are produced due to interaction of gravitational forces and density differences and Boussinesq approximation is yielded to notice their effects. Several studies have been conducted regarding thermosolutal diffusion in open configurations and under different physical circumstances which are referred in^3–6^. To be more specific, double diffusion naturally convective flow in enclosures have also been commenced like, Hyun and Lee^7,8^ analyzed influence of externally imposed assisting opposing diffusion gradients on double diffusion phenomenon in rectangular chamber. They also verified computed results by constructing comparison with experimental outcomes. Dual solution interpreting the impact of aiding and opposing buoyancy forces on thermal and solutal differences in vertical enclosure was performed by Mahapatra et al.^9^. Double diffusive transport in moist air saturated in a parallelogrammical enclosure for both combined situations of assisting and opposing buoyancy forces was studied by Costa^10^. Ghorayeb et al.^11^ performed comparative study to report analytical and numerical assessment about dually diffusive transport mechanism in power law fluid contained in shallow enclosure. Implication of magnetic field in controlling thermosolutal diffusion in viscous fluid enclosed in rectangular chamber along with provision of uniform heat source was manifested by Teamah^12^. Description about temperature distribution for opposing and assisting concentration gradients in flow of viscous fluid in rectangular enclosure was scrutinized by Abdallah et al.^13^. Illustration about thermosolutal diffusion in permeable media by measuring the impact of Darcy number on Nusselt and Sherwood number was manifested by Krishna et al.^14^. Azad et al.^15^ dealt with convectively driven flow of binary fluid confined in partially filled cavity immersed in permeable media.

Shapes of enclosure is highly essentials in creating diffusion due to the impact of buoyancy-forces generated by impact of gravitational forces. Gravity force always act in vertical direction to generate thermal and solutal gradients in regular shaped enclosures like square, rectangle, triangle and so many. In addition, alteration in boundaries of enclosure with corrugation execute more expressive change in respective gradients. In past ordinary enclosures were considered as a domain in order to investigate dynamical characteristics of liquids. But now a day, due to advancement in technology and scientific problems incorporation of complexly structured chambers. Applications of such domains are found in civil engineering (roofing and building structures), paint and emulsions industry, PVC pipes, double glazed windows and so many to mention. Alamiri et al.^16^ probed buoyantly induced flow of viscous fluid in square enclosure and attained variation in streamlines and isotherms against geometrical parameters. Soroush et al.^17^ delineated diffusion in rectangular enclosure under the combined effect of thermosolutal gradients. Convective transport resulted in view of thermosolutal gradients was computationally persuaded by Sourtiji et al.^18^. Influence of uniformed thermosolutal sources in generation of diffusive motion in inclined glazing enclosure was adumbrated by Manjunatha et al.^19^. Dually diffusive free convective flow of viscous liquid in partially heated square enclosure was explained by Nithyadevi and Yang^20^. Nosheen et al.^21^ employed computational method for simulations to analyze behavior of magnetized naturally convective fluid flow in cavity. Zeeshan et al.^22,23^ emphasized on motion of gyrotactic microorganisms in non-Newtonian liquid in wavy channel by implementing finite differencing method. Mansour et al.^24^ presented numerical investigation on magnetically influenced convective-flow of nanofluid in an enclosure with physical factors. Shamshuddin et al.^25^ conducted an analysis of heat and mass transfer in a variable hydromagnetic flow involving Copper and Copper oxide–water nanoparticles suspended in a nanofluid. This analysis was performed on a non-linear radially stretching surfaces, considering the influence of various factors. In their work, Al-Kouz et al.^26^ conducted a numerical analysis of thermal convection resulting from buoyancy forces within a conical annular porous-gap. This annular space was oriented vertically and included a discrete heat source, while being filled with a nanoliquid was subjected to the influence of a Lorentz force. In their study^27^, conducted empirical parametric research to examine natural convection and the formation of entropy within a cavity that was filled with a nanofluid. This cavity was exposed to a magnetic field and included the presence of porous medium. Rashad et al.^28^ divulged convection in nanofluid flow in a trapezoidal lid driven cavity along with the presence of discrete heat sources. Rashad et al.^29^ examined free convective magnetized nanoliquid flow in an inclined porous cavity. In the study conducted by Nilankush^30^, the focus was on the computational analysis of a magnetized Ag-MgO water hybrid nanofluid flow within a cube shaped cavity containing an inner circular cylinder. The research discussed in^31^ provided insight into the hydrothermal conduction and entropy analysis of a magnetized Ag-MgO water hybrid nanofluid flowing through an octagonal cavity and a circular cylinder. Additionally, rectangular heated fins are affixed to the inner hot cylinder. Nilankush et al.^32^ elucidated hydrothermal changes in alumina-water nanofluid flow in partially heated hexagonal enclosure containing parallel fins. Inside this hexagonal compartment three parallels fins were strategically placed. Some latest studies related to hybrid nanofluid flows in confined geometries are addressed in Refs.^33–35^.

Through a comprehensive review of the existing literature, it becomes evident that studies on convective diffusion have predominantly focused on thermal aspects. Particularly, significant research has been conducted on convective heat transfer in nanofluids containing single nanoparticles. However, a significant gap is observed in the understanding of thermal and solutal convective transport in hybrid nanofluids. Limited attention has been given to investigating this area, highlighting the need for further research in this direction. In spite of recommendable need of hybrid nanomaterials in different thermal exchange processes still huge focus by researchers is required. So, the intend behind the deliverance of present study is to fill this space by analyzing flow and thermal attributes of ordinary base liquid by adding hybrid nanoparticles to meet desirable demand managed in different phenomenon. For this purpose, a corrugated enclosure is taken into account by filling with water and adding Cu–Al_2_O_3_ hybridized nanoparticles. We have used Cu–Al_2_O_3_ nanoparticles because Cu nanoparticles are known for their excellent electrical and thermal conductivity. This makes them suitable for application in electronics, conductive inks, and heat transfer materials. Secondly, Al_2_O_3_ nanoparticles are lightweight and have good insulating properties. They are used in application where electrical insulation or lightweight material are required, such as in the aerospace industry. Magnetic field is also employed to make present work more significant. Combined heat and mass diffusions are also examined in enclosure by placing circular cylinder with provision of uniform temperature and concentration distributions. Novelties of current work are mentioned as below:

1. Investigation of thermosolutal diffusion in complex domain (inclined wall corrugation)
2. Induction of hybrid nanoparticles (Cu-Al_2_O_3_) to adumbrate dual (heat and mass) diffusion in domain
3. Implementation of magnetic field to notice its impact on heat and mass fields.
4. Installation of uniformly heated and concentrated circular cylinder.

To the best of authors knowledge presently conducted work has not yet been considered and it will definitely provide motivation to researcher to work in this direction.

## Mathematical formulation

**Figure 1:**
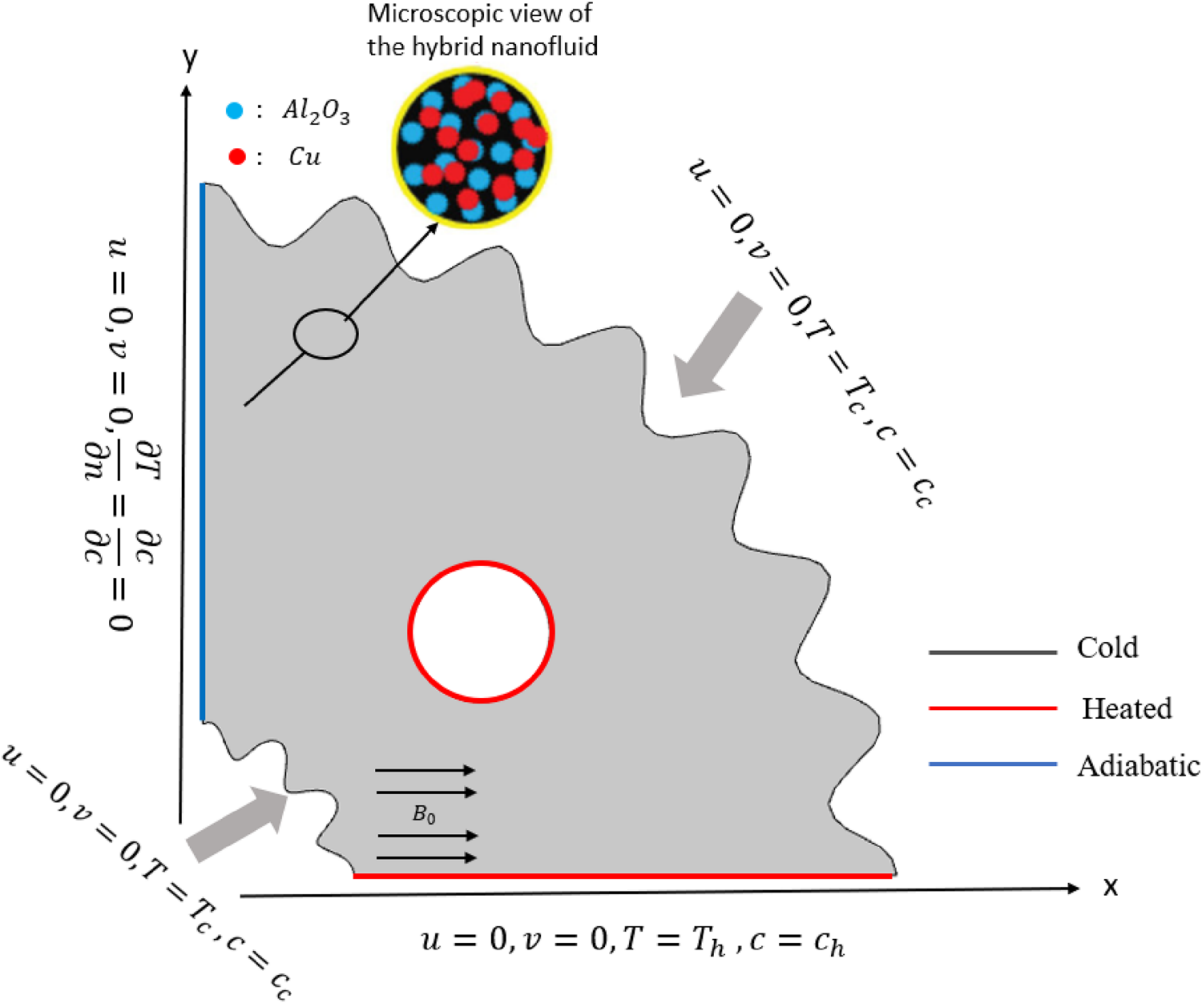
Graphical visualization of domain.

Geometrical interpretation of work is delineated in Fig. 1 which comprises of square cavity with curved surfaces and circular heated cylindrical obstacle is placed inside the enclosure. Cu–Al_2_O_3_–water hybrid nanofluid. Additionally, an external magnetic field with a strength of ${(B}_{0})$ is employed in horizontal direction. Equivalent amounts of copper and alumina-nanoparticles (50% of Cu nanoparticles + 50% of Al_2_O_3_) is dissolved in water to form hybrid nanofluid. The physical characteristics of a working hybrid nanofluid are assumed to be constant under the Boussinesq assumption with the exception of density variations.

## Modelling of problem

Governing equations in dimensional form describing 2D, steady double diffusive natural convective flow of an incompressible electrically conducting hybrid nanofluid are represented as below (see Ref.^36^)

1 $$\frac{\partial u}{\partial {\text{x}}} + \frac{\partial v}{\partial {\text{y}}}=0,$$

2 $$u\frac{\partial u}{\partial {\text{x}}}+v\frac{\partial u}{\partial {\text{y}}}=-\frac{1}{{\rho }_{hnf}}\frac{\partial p}{\partial {\text{x}}}+\frac{{\mu }_{hnf}}{{\rho }_{hnf}}\left(\frac{{\partial }^{2}u}{\partial {x}^{2}}+\frac{{\partial }^{2}u}{\partial {y}^{2}}\right),$$

3 $$u\frac{\partial v}{\partial {\text{x}}}+v\frac{\partial v}{\partial {\text{y}}}=-\frac{1}{{\rho }_{hnf}}\frac{\partial p}{\partial {\text{y}}}+\frac{{\mu }_{hnf}}{{\rho }_{hnf}}\left(\frac{{\partial }^{2}v}{\partial {x}^{2}}+\frac{{\partial }^{2}v}{\partial {y}^{2}}\right)+g\frac{{\left(\rho {\beta }_{T}\right)}_{hnf}}{{\rho }_{hnf}}\left(T-{T}_{c}\right)+g\frac{{\left(\rho {\beta }_{c}\right)}_{hnf}}{{\rho }_{hnf}}\left(c-{c}_{c}\right)-\frac{{\sigma }_{hnf}}{{\rho }_{hnf}}{B}_{0}^{2}v,$$

4 $$u\frac{\partial T}{\partial {\text{x}}}+v\frac{\partial T}{\partial {\text{y}}}={\alpha }_{hnf}\left(\frac{{\partial }^{2}T}{\partial {x}^{2}}+\frac{{\partial }^{2}T}{\partial {y}^{2}}\right),$$

5 $$u\frac{\partial c}{\partial {\text{x}}}+v\frac{\partial c}{\partial {\text{y}}}=D\left(\frac{{\partial }^{2}c}{\partial {x}^{2}}+\frac{{\partial }^{2}c}{\partial {y}^{2}}\right).$$

where $(u,v)$ are the components of velocity along the $(x,y)$, $T$ and $p$ are the temperature and pressure of the hybrid nanofluid.

Numerous formulations addressing the thermophysical properties of hybrid nanofluid have been introduced in scientific literature. In this particular study, we have employed the physical parameters for hybrid nanofluid as provided by Takabi and Salehi^36^, which are documented in Table 1.

**Table 1:** Properties of hybrid nanofluid^36^.

Hybrid-nanofluid characteristics	Applied relation
Nanoparticles concentration:	$\phi ={\phi }_{C}+{\phi }_{A}$
Density:	${\rho }_{hnf}=\left(1-\phi \right){\rho }_{f}+{\phi }_{A}{\rho }_{A}+{\phi }_{C}{\rho }_{C}$
Dynamic viscosity:	${\mu }_{hnf}=\frac{{\mu }_{f} }{{(1-\phi )}^{2.5}}$
Thermal conductivity:	$\frac{{k}_{hnf}}{{k}_{f}}=\frac{\frac{{\phi }_{A}{k}_{A}+{\phi }_{C}{k}_{C }}{\phi }+2{k}_{f}+2\left({\phi }_{A}{k}_{A}+{\phi }_{C}{k}_{C }\right)-2\left({\phi }_{A}+{\phi }_{C}\right){k}_{f}}{\frac{{\phi }_{A}{k}_{A}+{\phi }_{C}{k}_{C }}{\phi }+2{k}_{f}-\left({\phi }_{A}{k}_{A}+{\phi }_{C}{k}_{C }\right)+\left({\phi }_{A}+{\phi }_{C}\right){k}_{f}}$
Heat capacity:	${\left(\rho {C}_{P}\right)}_{hnf}=\left(1-\phi \right){\left(\rho {C}_{P}\right)}_{f}+{\phi }_{A}{\left(\rho {C}_{P}\right)}_{A}+{\phi }_{C}{\left(\rho {C}_{P}\right)}_{C}$
Thermal expansion co-efficient:	${\left(\beta \right)}_{hnf}=\left(1-\phi \right){\left(\beta \right)}_{f}+{\phi }_{A}{\left(\beta \right)}_{A}+{\phi }_{C}{\left(\beta \right)}_{C}$
Thermal diffusivity:	${\alpha }_{hnf}=\frac{{k}_{hnf}}{{\left(\rho {C}_{P}\right)}_{hnf}}$
Electrical conductivity:	$\frac{{\sigma }_{nf}}{{\sigma }_{f}}=\frac{\frac{{\phi }_{A}{\sigma }_{A}+{\phi }_{C}{\sigma }_{C }}{\phi }+2{\sigma }_{f}+2\left({\phi }_{A}{\sigma }_{A}+{\phi }_{C}{\sigma }_{C }\right)-2\left({\phi }_{A}+{\phi }_{C}\right){\sigma }_{f}}{\frac{{\phi }_{A}{\sigma }_{A}+{\phi }_{C}{\sigma }_{C }}{\phi }+2{\sigma }_{f}-\left({\phi }_{A}{\sigma }_{A}+{\phi }_{C}{\sigma }_{C }\right)+\left({\phi }_{A}+{\phi }_{C}\right){\sigma }_{f}}.$

In terms of dimensions, the associated boundary conditions are represented as follows.

6 $$u=0, v=0, T={T}_{h}, c={c}_{h} (\mathrm{Hot side}),$$

7 $$u=0, v=0, T={T}_{c}, c={c}_{c} (\mathrm{Cold side}),$$

8 $$u=0, v=0, \frac{\partial T}{\partial n}=\frac{\partial c}{\partial n}=0, (\mathrm{Remaining walls}).$$

Here, *n* highlights the normal vector on the boundary.

We employ the similarity transformation described in Ref.^37^ to transform Eqs. (1–5) along with BCs defined in Eqs. (6–8) into a non-dimensional form.

9 $$\left({X}^{*},{Y}^{*}\right)=\frac{\left(x,y\right)}{L}, \left({U}^{*},{V}^{*}\right)=\frac{\left(u,v\right)L}{{\alpha }_{f}}, {P}^{*}=\frac{p{L}^{2}}{{\rho }_{f}{{\alpha }_{f}}^{2}} , {\theta }^{*}=\frac{T-{T}_{c}}{{T}_{h}-{T}_{c}}, {C}^{*}=\frac{c-{c}_{c}}{{c}_{h}-{c}_{c}} .$$

The dimensionless form of the continuity, momentum, and energy equations are as follows after applying similarity transformation.

10 $$\frac{\partial {U}^{*}}{\partial {X}^{*}}+\frac{\partial {V}^{*}}{\partial {Y}^{*}}=0,$$

11 $$\left({U}^{*}\frac{\partial {U}^{*}}{\partial {X}^{*}}+{V}^{*}\frac{\partial {U}^{*}}{\partial {Y}^{*}}\right)=-\frac{{\rho }_{f}}{{\rho }_{hnf}}\frac{\partial {P}^{*}}{\partial {X}^{*}}+\frac{\frac{{\mu }_{hnf}}{{\mu }_{f}}}{\frac{{\rho }_{hnf}}{{\rho }_{f}}}\left(\frac{{\partial }^{2}{U}^{*}}{{\partial }^{2}{X}^{*}}+\frac{{\partial }^{2}{U}^{*}}{{\partial }^{2}{Y}^{*}}\right),$$

12 $$ \begin{gathered} \left( {U^{*} \frac{{\partial V^{*} }}{{\partial X^{*} }} + V^{*} \frac{{\partial V^{*} }}{{\partial Y^{*} }}} \right) = - \frac{{\rho_{f} }}{{\rho_{hnf} }}\frac{{\partial P^{*} }}{{\partial Y^{*} }} + \frac{{\frac{{\mu_{hnf} }}{{\mu_{f} }}}}{{\frac{{\rho_{hnf} }}{{\rho_{f} }}}}\left( {\frac{{\partial^{2} V^{*} }}{{\partial^{2} X^{*} }} + \frac{{\partial^{2} V^{*} }}{{\partial^{2} Y^{*} }}} \right) \hfill \\ \frac{{\left( {\rho \beta } \right)_{hnf} }}{{\rho_{hnf} \beta_{f} }} Ra {\text{Pr }}\left( {\theta^{*} + NC^{*} } \right) - \frac{{\frac{{\sigma_{hnf} }}{{\sigma_{f} }}}}{{\frac{{\rho_{hnf} }}{{\rho_{f} }}}}Ha^{2} Pr V^{*} , \hfill \\ \end{gathered} $$

13 $${U}^{*}\frac{\partial {\theta }^{*}}{\partial {X}^{*}}+{V}^{*}\frac{\partial {\theta }^{*}}{\partial {Y}^{*}}=\frac{\frac{{k}_{hnf}}{{k}_{f}}}{ \frac{{({\rho C}_{p})}_{hnf}}{{({\rho C}_{p})}_{f}}}\left(\frac{{\partial }^{2}{\theta }^{*}}{\partial {{X}^{*}}^{2}}+\frac{{\partial }^{2}{\theta }^{*}}{\partial {{Y}^{*}}^{2}}\right),$$

14 $${U}^{*}\frac{\partial {C}^{*}}{\partial {X}^{*}}+{V}^{*}\frac{\partial {C}^{*}}{\partial {Y}^{*}}=\frac{1}{Le}\left(\frac{{\partial }^{2}{C}^{*}}{\partial {{X}^{*}}^{2}}+\frac{{\partial }^{2}{C}^{*}}{\partial {{Y}^{*}}^{2}}\right).$$

The BCs in non-dimensional form are:

15 $${U}^{*}=0, {V}^{*}=0, {\theta }^{*}=1,{ C}^{*}=1 (\mathrm{Hot side}),$$

16 $${U}^{*}=0, {V}^{*}=0, {\theta }^{*}=0,{ C}^{*}=0 (\mathrm{cold side}),$$

17 $${U}^{*}=0, {V}^{*}=0, \frac{\partial {\theta }^{*}}{\partial n}=\frac{\partial {C}^{*}}{\partial n}=0 (\mathrm{Remaining walls}).$$

The physical parameters implicated in Eqs. (10–14) are defined as follow:

18 $$Pr=\frac{{v}_{f}}{{\alpha }_{f}}, Ra=\frac{g{\beta }_{T}({T}_{h}-{T}_{c}{)L}^{3}}{{\alpha }_{f}{v}_{f}}, Ha={B}_{0}L\sqrt{\frac{{\sigma }_{f}}{{\rho }_{f}{v}_{f}}}, Le=\frac{{\alpha }_{f}}{D}.$$

where the Hartmann number (Ha) illustrates the effects of magnetic forces, Rayleigh number (Ra) demonstrates the impact of buoyancy forces. The local Nusselt numbers, average Nusselt numbers, local Sherwood number and, average Sherwood number at the heated wall of the enclosure can be stated as follows;

19 $${Nu}_{local}=-\frac{{k}_{hnf}}{{k}_{f}}\frac{\partial {\theta }^{*}}{\partial n}, {\text{and}} {Sh}_{local}=-\frac{\partial {C}^{*}}{\partial n}$$

20 $$N{u}_{avg}={\int }_{S}^{1}{Nu}_{local} dX, and S{h}_{avg}={\int }_{S}^{1}{Sh}_{local} dX.$$

### Computational procedure

Solution of engineering problems modelled mathematically is attained by two primary approaches i.e. analytical and numerical. Analytical methods provide solution for simple problem because they involve mathematical relations developed for flow situations. On contrary to it, numerical methods have advantages that they provide solution at discrete data points in the domain which are more realistic for implementation in real world processes. Likewise, physical insight of problems in regular domains can be easily accessed through analytical approaches whereas, computational approaches play vital role in attaining feasible outcomes from complexly structured units. Specifically, FEM is typically considered to be the most adaptable and well suited for complicated geometries. For this purpose, COMSOL^38^ Multiphysics software based on finite element scheme is used for numerical simulation. COMSOL has many advantages, including its user-friendly interface wide range of capabilities and large user community. However, it also has some disadvantages including its steep learning curve high cost and limited compatibility with other packages.

**Figure 2:**
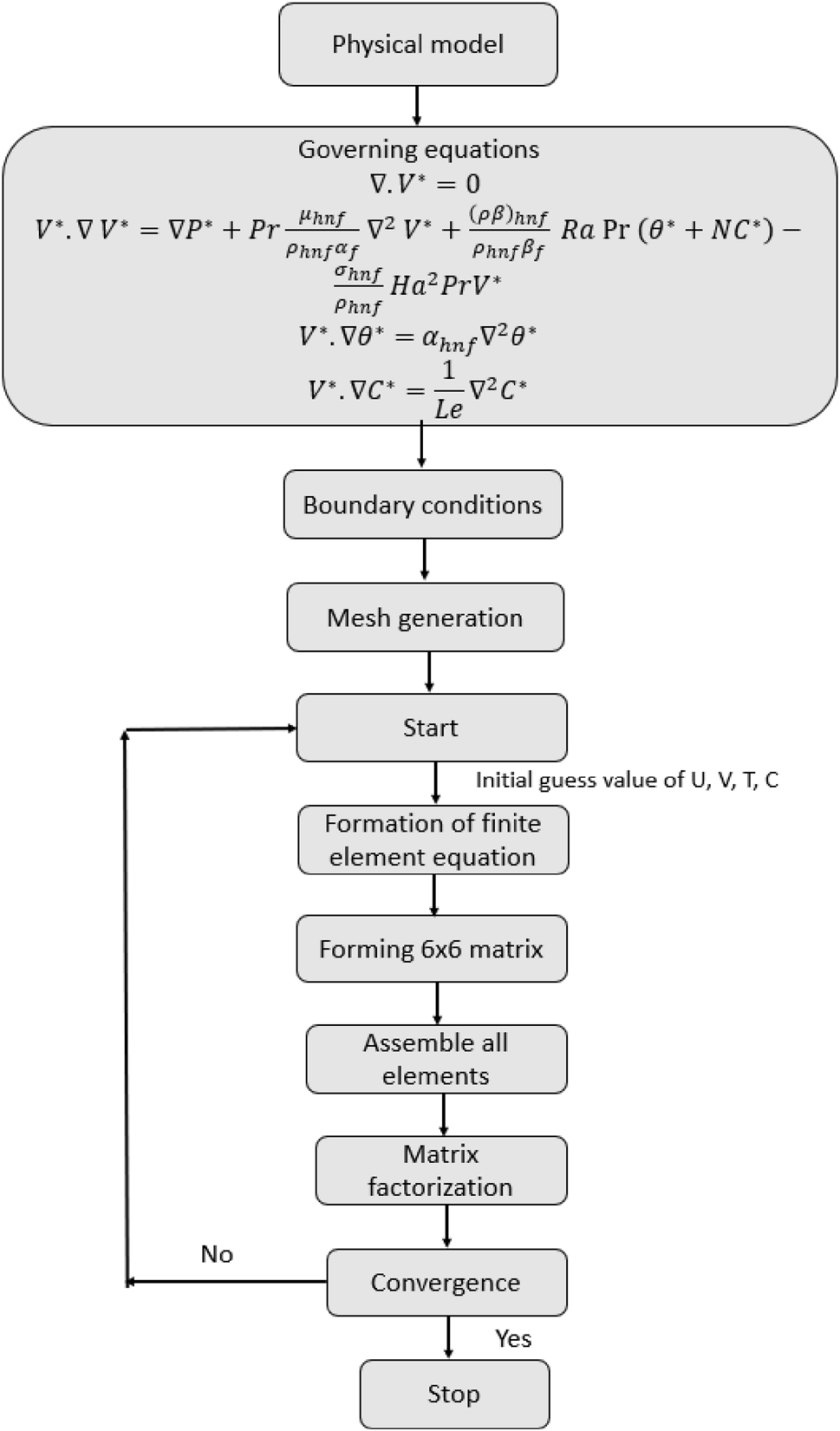
Flow chart of the FEM.

It enables a more accurate discretization of computational domain by distributing it into rectangular and triangular element with execution of hp-refinement. Afterwards, Lagrange interpolation formula is capitalized to produce shape function that defines the behavior of field variables at each node. Currently, quadratic shape functions are used to approximate velocity and temperature fields whereas pressure is estimated by linear shape functions. After discretization of domain, equations at element level are constructed by employing weak formulation and local stiffness matrices are generated which are at last combined to form global matrix for whole domain. Following that, Newton's technique is applied to linearize non-linearized expressions, and the linear system of equations that is produced as a consequence is solved directly by employing elimination-based method solver renowned as PARDISO. Steps involved in the process of computations are shown in Fig. 2. The following convergence condition is established for the iterations using the nonlinear function $\left|\frac{{\chi }^{n+1}-{\chi }^{n}}{{\chi }^{n+1}}\right|<1{0}^{-6}$.

**Figure 3:**
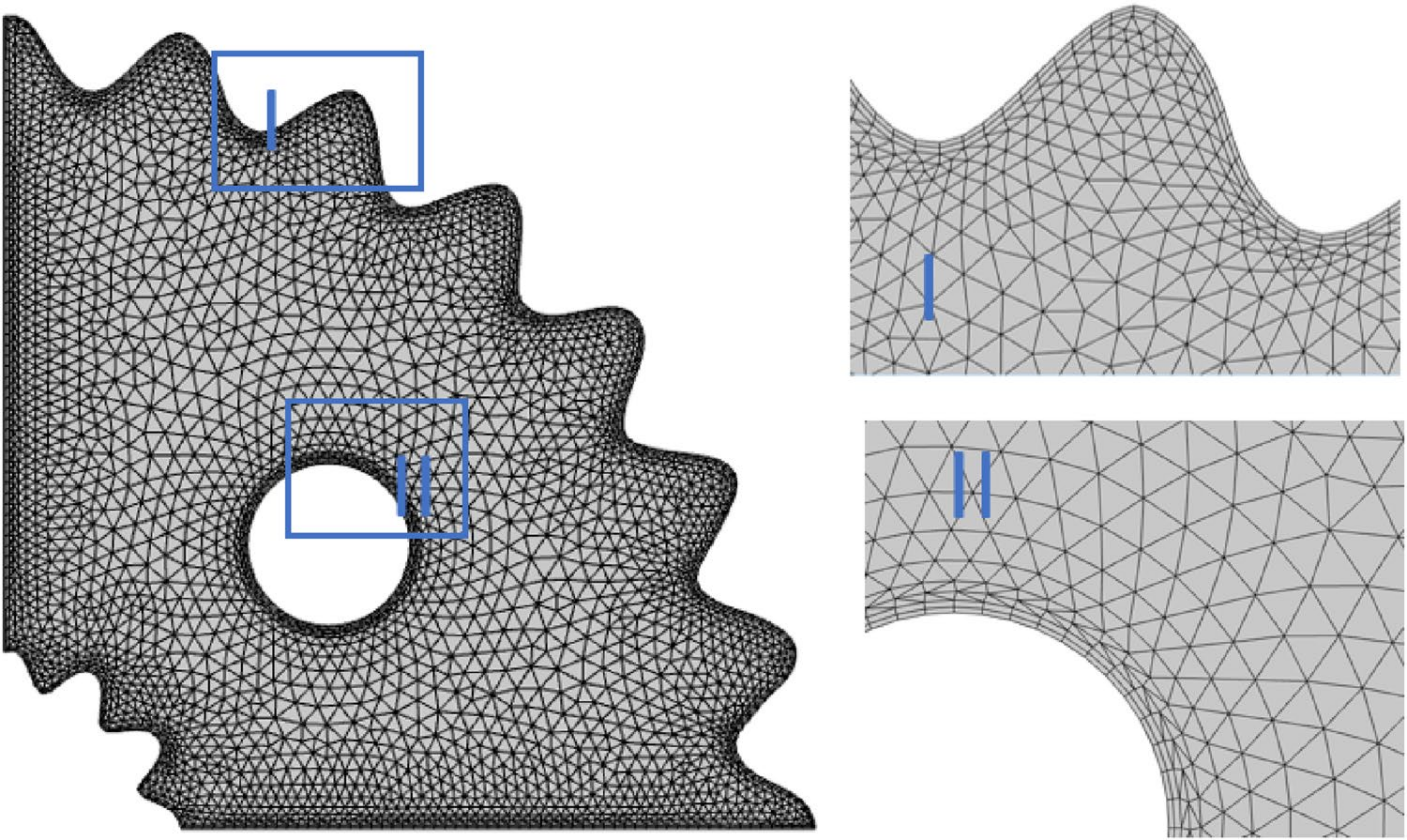
Mesh generation of the wavy cavity.

Figure 3 displays the finer computational grid containing rectangular elements at boundaries and triangular elements in the internal space. Table 2 illustrates the number of elements and degrees of freedom across various refinement levels.

**Table 2:** Grid sensitivity test at *Pr* = 6.2, *N* = *1*, *Ha* = 25*,* ${Ra=10}^{4},$ *Le* = *2.5* and $\phi =0.04.$

Mesh (element)	DOFs	$N{u}_{avg}$
L1 (1646)	14,725	10.837
L2 (2403)	22,487	11.132
L3 (6440)	53,416	12.160
L4 (17,081)	155,739	13.063
L5 (23,527)	191,372	13.063

### Grid sensitivity test

**Figure 4:**
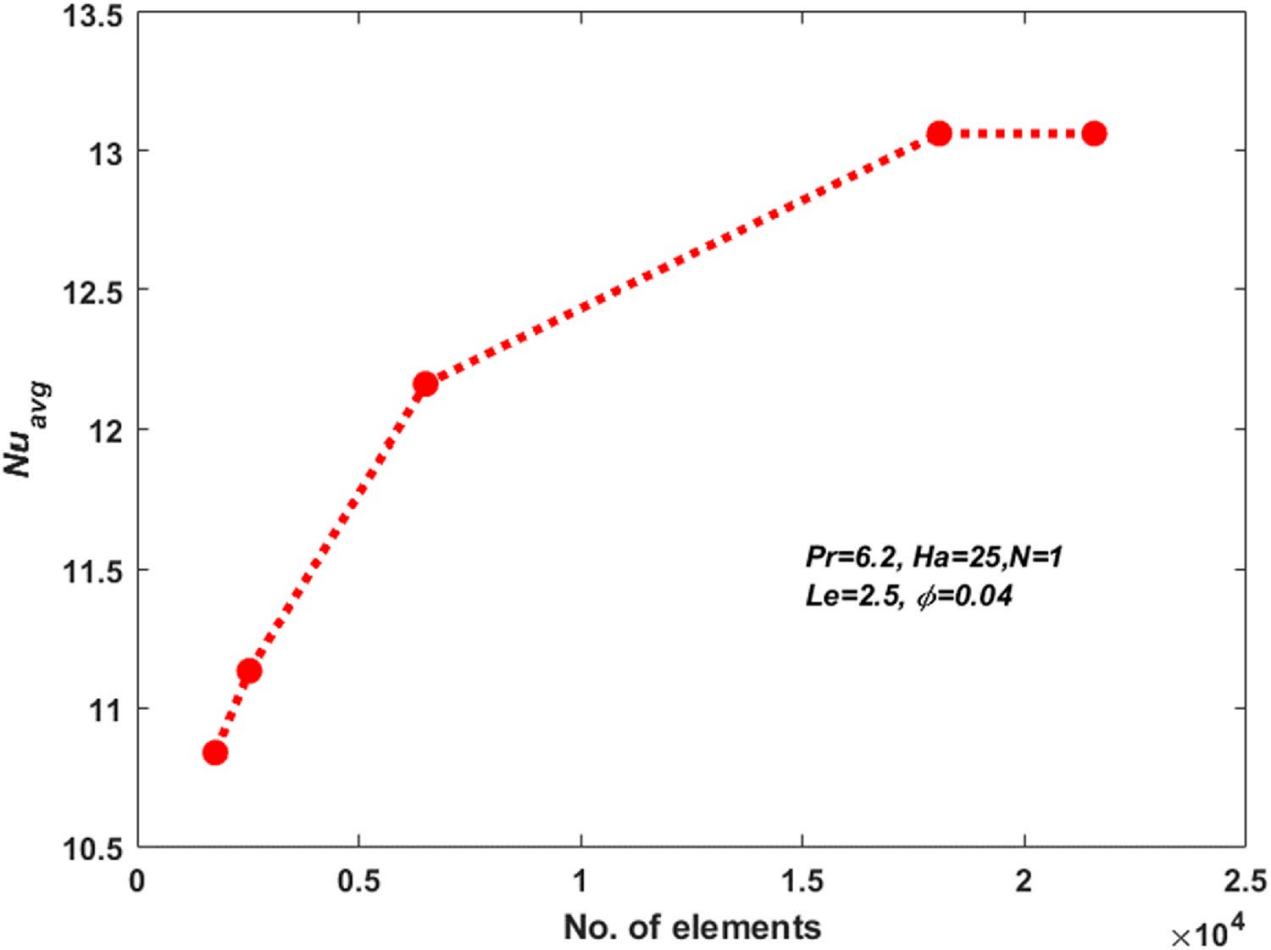
Grid refinement test.

Examining variation in mesh size, we obtained the numerical solution to assess the influence of mesh size on the results. Five different meshes were examined for this purpose. The average Nusselt number values around the hot inner cylinder was determined for a grid refinement test, as shown in Table 2 and Fig. 4. Table 2 reveals that increasing the number off mesh configurations have minimal effects on the average Nusselt number, which can be considered negligible. Consequently, it is noted that a mesh size consisting of 18,080 elements yields a satisfactory solution for this investigation.

### Validations of computational procedure

**Figure 5:**
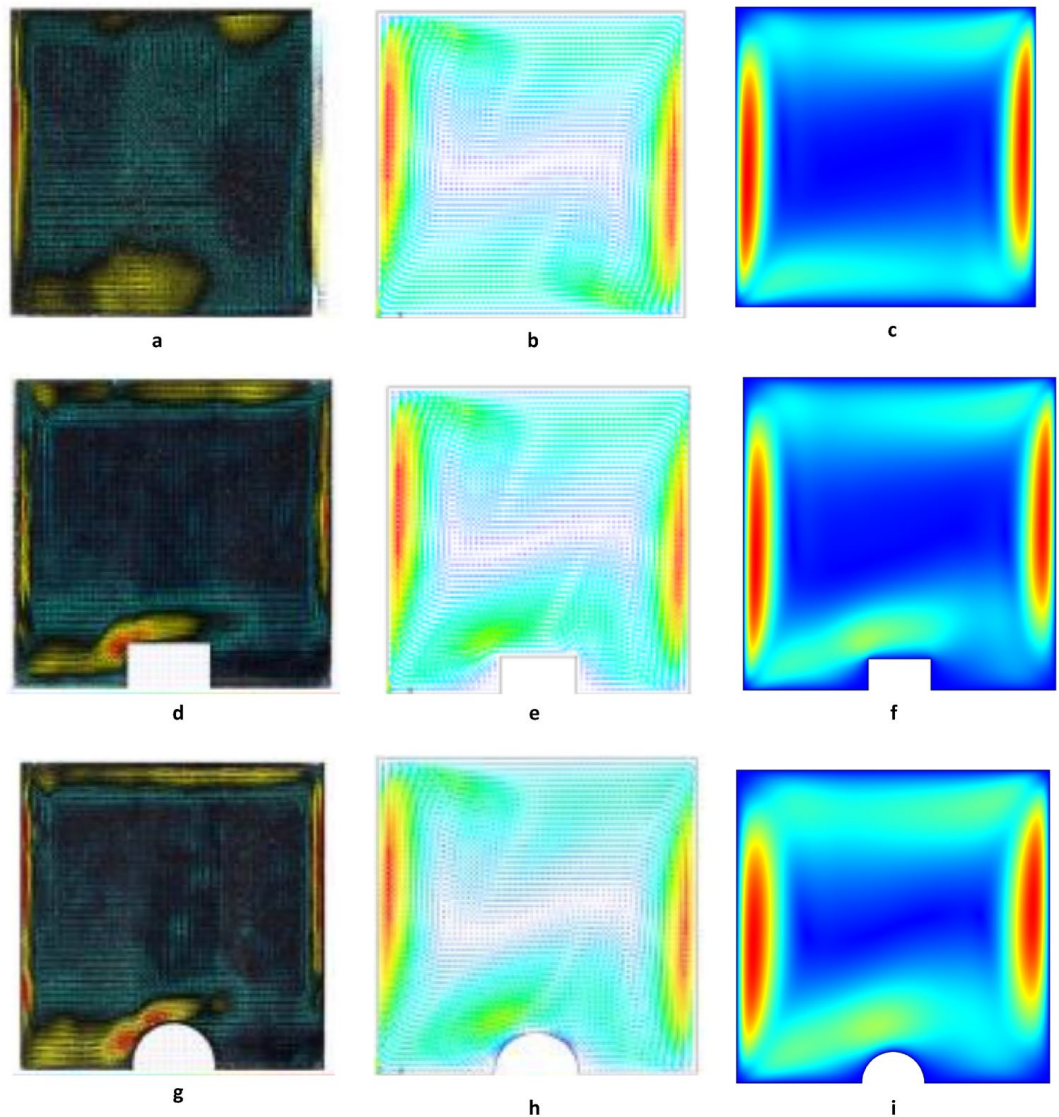
(**a**–**i**) Comparison of velocity field of current study with Lizardi et al.^40^. (**a**) Experimental velocity field without protuberance by Lizardi et al.^40^. (**b**) Numerical velocity field without protuberance by Lizardi et al.^40^. (**c**) Numerical velocity field without protuberance, present work. (**d**) Experimental velocity field with rectangular protuberance by Lizardi et al.^40^. (**e**) Numerical velocity field with rectangular protuberance by Lizardi et al.^40^. (**f**) Numerical velocity field with rectangular protuberance, present work. (**g**) Experimental velocity field with semi-circular protuberance by Lizardi et al.^40^. (**h**) Numerical velocity field with semi-circular protuberance by Lizardi et al.^40^. (**i**) Numerical velocity field with semi-circular protuberance, present work.

**Figure 6:**
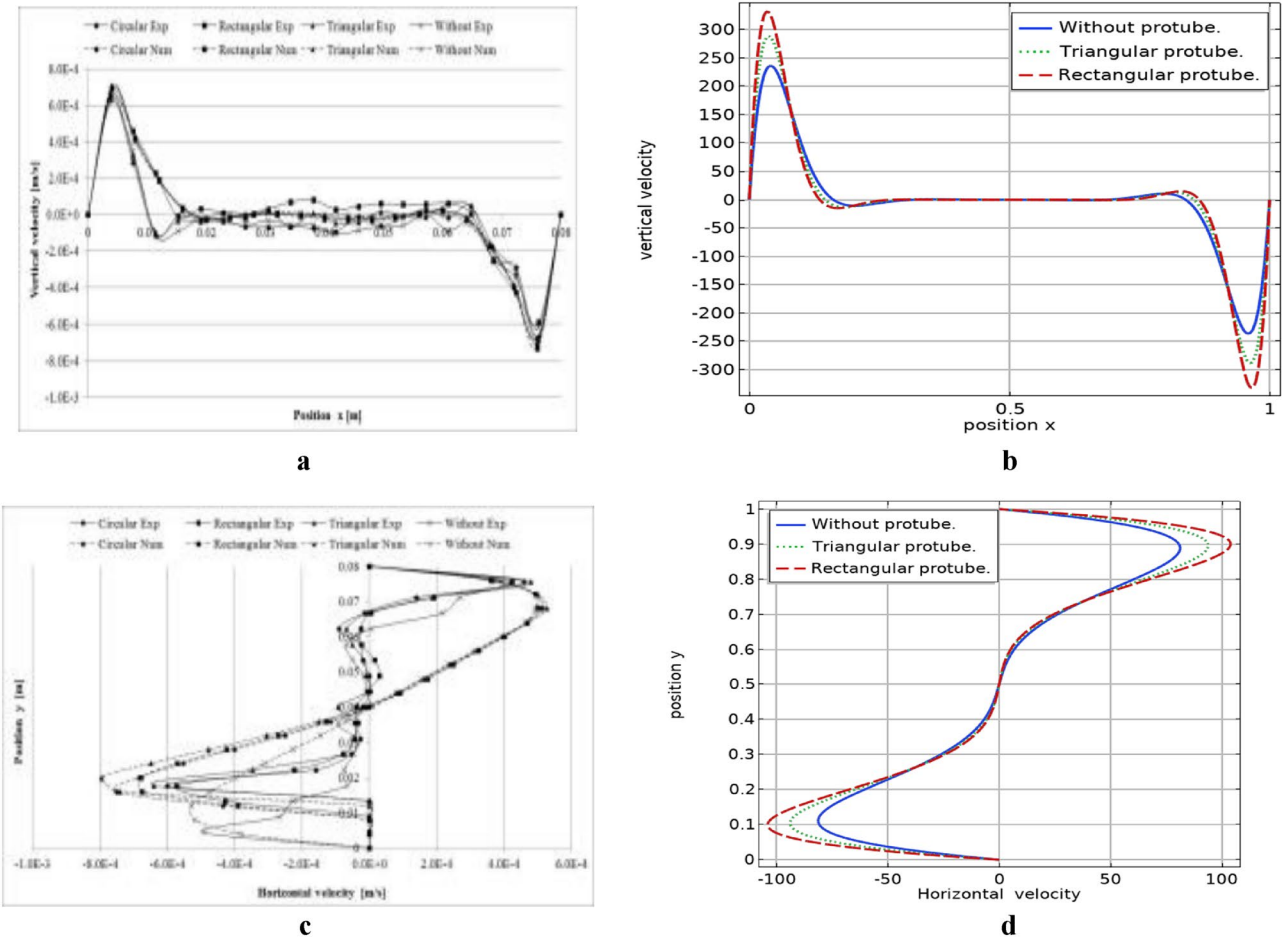
(**a**–**d**) Comparison of vertical and horizontal velocity distribution of current study with Lizardi et al.^40^. (**a**) Distribution of vertical velocity for the $y=0.04$ position by Lizardi et al.^40^. (**b**) Distribution of vertical velocity for the $y=0.04$ position, present work. (**c**) Distribution of horizontal velocity for the $x=0.04$ position by Lizardi et al.^40^. (**d**) Distribution of horizontal velocity for the $x=0.04$ position, present work.

To ensure numerical accuracy the current model has performed a validation test. The performance off the numerical model has been compared with the finite element-based code results of Shafqat et al.^39^ for natural convection of viscous fluid in staggered cavity. The average Nusselt number was examined for $N=1, Le=2.5, Ha=20$ and $Pr=6.2$ and for various values of Ra. As shown, Table 3 shows that the results are in good agreement with previous results. To enhance the analysis conducted in this study the developed solver is also validated using the experimental findings of Lizardi et al.^40^ regarding natural convection. The validation results are presented in Table 4, demonstrating a favorable agreement between the proposed model and the experimental outcomes. Further validation of the current work is conducted by comparing the experimental and numerical velocity fields as well as the horizontal and vertical velocity distributions presented by Lizardi et al.^40^. A comprehensive analysis presenting agreement between experimental and numerical results for velocity field in the presence and absence of protuberance is compared and displayed in Fig. 5a–i. In addition, Fig. 6a–d reveals a significant resemblance between horizontal and vertical components of velocity by drawing cutlines in the presence and absence of protuberance. From the displayed sketches, complete agreement between present and existing simulations documented by Lizardi et al.^40^ by both experimental and numerical studies.

**Table 3:** Comparison of current and previous results for the average Nusselt number.

*Ra*	Present work	Shafqat et al.^39^
${10}^{4}$	1.26628	1.27095
${10}^{5}$	3.85450	3.81194
${10}^{6}$	8.57565	8.54480
${10}^{7}$	16.86370	16.86152

**Table 4:** Comparison of current model with experimental data of Lizardi et al.^40^.

$${Nu}_{avg}$$		
Experimental result^40^	Numerical result^40^	Present result
13.21	13.90	13.87

## Results and interpretation

In this paper, we investigated magnetically influenced thermosolutal free convection flow of Cu–Al_2_O_3_–H_2_O hybrid nanofluid in a corrugated enclosure emplacing a heated circular cylinder. The enclosure is heated and concentrated from the lower wall, while the corrugated surfaces are kept at a lower temperature and concentration. In this study, it is crucial to acknowledge that gravitational forces align nearly parallel to the thermal gradient. This sets the stage for a nuanced interplay between the weight of the fluid and buoyancy forces. Unlike scenarios where gravitational forces align parallel to the heat source, the dynamics here involve a distinctive competition. Gravity seeks to impede fluid movement, while buoyancy forces endeavor to instigate circulation, aligned with the temperature gradient. This intricate relationship is extensively explored in our previous work^41,42^. The impact of various physical parameters like Rayleigh number ($${10}^{4}$$ ≤ *Ra* ≤ $${10}^{6})$$, Hartmann number $$(0$$ ≤ *Ha* ≤ $$100)$$,buoyancy ratio $$(1$$ ≤ *N* ≤ $$3)$$, Lewis number $$(0.1$$ ≤ *Le* ≤ $$10)$$, and hybrid nanofluid volume fraction $$(0$$ ≤ $$\phi $$ ≤ $$0.05)$$ are examined in term of streamlines, isotherms, isoconcentration, average Nusselt number and average Sherwood number.

### Effects of Rayleigh number

**Figure 7:**
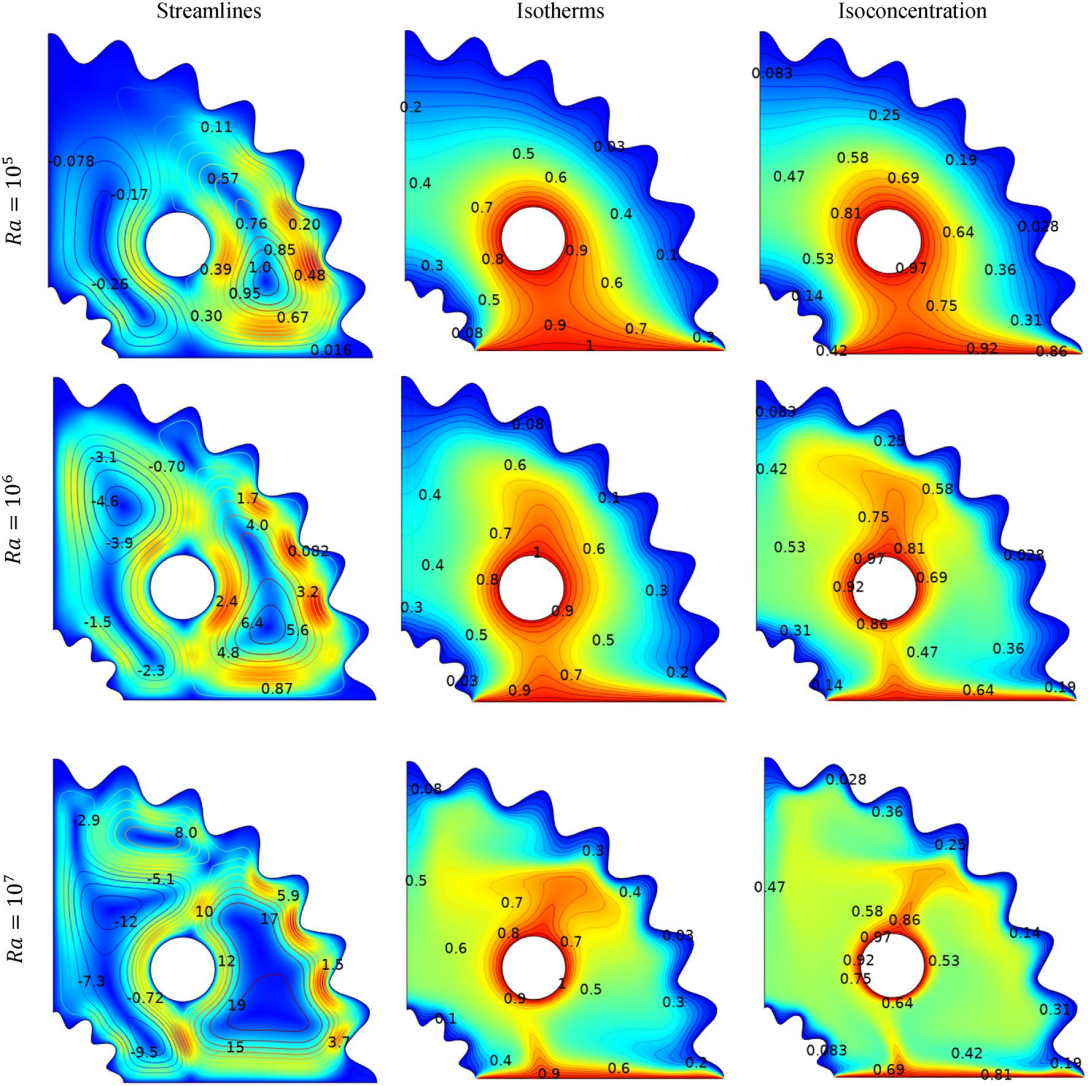
Impact of $Ra$ on streamlines, isotherm and isoconcentration.

The impact of Rayleigh number (Ra) on streamlines, isotherms, and isoconcentrations is represented in Fig. 7. The depicted sketches reveal that by increasing, $(Ra)$ the magnitude of velocity increase. As the (Ra) number increases, the temperature difference between the heated and cold walls also increases, causing density variations in the fluid within the cavity. Consequently, the fluid circulates more strongly, leading to an increase in the vortex within the cavity. It is also observed that the fluid moves up ward near the middle of the cavity due to buoyancy-force caused by the imposed thermal condition, and bifurcates due to the presence of solid cylinder, and then tends to drop near the vicinity of the cold wavy wall. As a result, two primaries symmetric circulations arise beside the centered circular cylinder, one of which is rotated clockwise (indicated by negative sign of values) and the other in the opposite direction. When $(Ra)$ is increased from ${10}^{5}$, ${10}^{6}$, to ${10}^{7}$, the maximum absolute stream function strengthens by 0.39, 2.439, and 12.103 respectively, a similar behaviour is observed in ref.^43^. As the heat source is in the centre of the corrugated and in the lower wall, temperature and concentration patterns are parallel near the heat sources at low ($Ra={10}^{5}$), indicating conduction dominant flow. At low (Ra), the effect of buoyancy is weak compared to viscous forces and the streamlines in the fluid tend to be mostly parallel and uniform, As the Ra increases, the buoyancy driven convection becomes dominants and streamlines become curved and start forming convection cell. The increase in $(Ra)$ causes a significant change in isotherms and isoconcentrations. In low Ra regime, the temperature and concentration distribution tend to be relatively uniform and isotherms are approximately parallel to each other. With increasing (Ra), the temperature distribution become highly non-uniform and isotherms are no longer parallel but curve.

### Effects of Hartmann number

**Figure 8:**
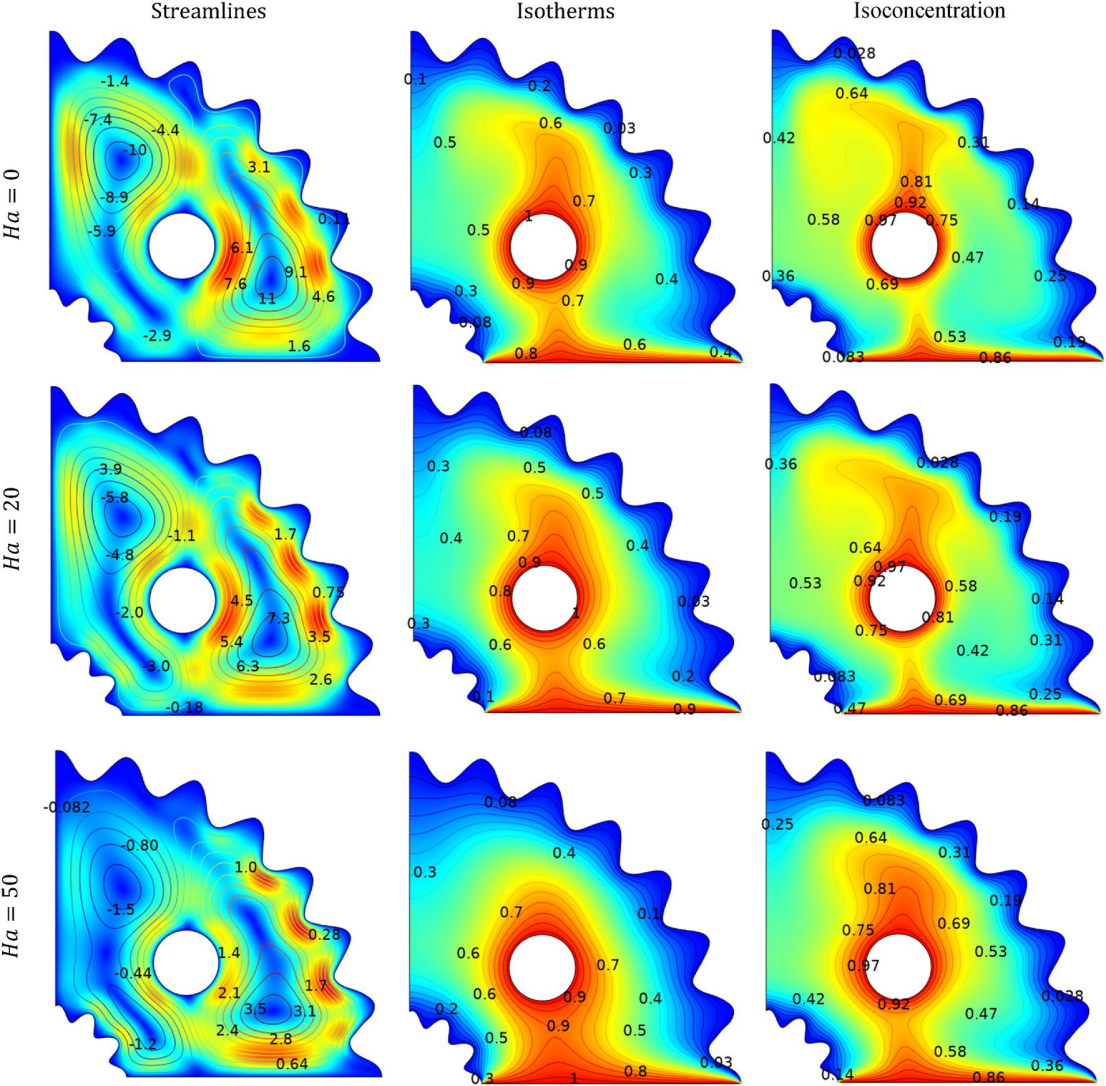
Impact of $Ha$ on streamlines, isotherms and isoconcentration.

Figure 8 represents the change in velocity, temperature, and isoconcentration distribution as a function of magnetic field parameter (*Ha)* at fixed value of *Pr* = 6.2, *N* = *1*, *Ra* = ${10}^{5}$, *Le* = *2.5* and $\phi =0.04$. Since the Hartmann number is implicated in the current investigation due to the inclusion of a magnetic field, which plays a function in decreasing the velocity profile and making. The flow regime laminar. In absence of magnetic field $\left(Ha=0\right)$ the value of stream function, ${|\psi |}_{max}=11$, which shows stronger convection effect. The velocity profile experiences a slight decrease with an increase in the magnetic field, same behaviour is observed in Ref.^44^. This is attributed to the heightened magnetic field generating Lorentz forces that counteract motion, thereby slowing it down. This observation is depicted in Fig. 8. The gradient of temperature contours decreases minutely with increasing (*Ha*), indicating that an increase in magnetic field effect leads to a domination of conduction. As a result, the magnetic field has little effect on the distribution of isotherms, and convection becomes less prominent at larger magnetic effects. Since the energy and mass transport equations are identical, the isoconcentration contour exhibit a behaviour similar to that of the isotherms contour, as illustrated in Fig. 8.

### Effects of nanoparticle volume fraction

**Figure 9:**
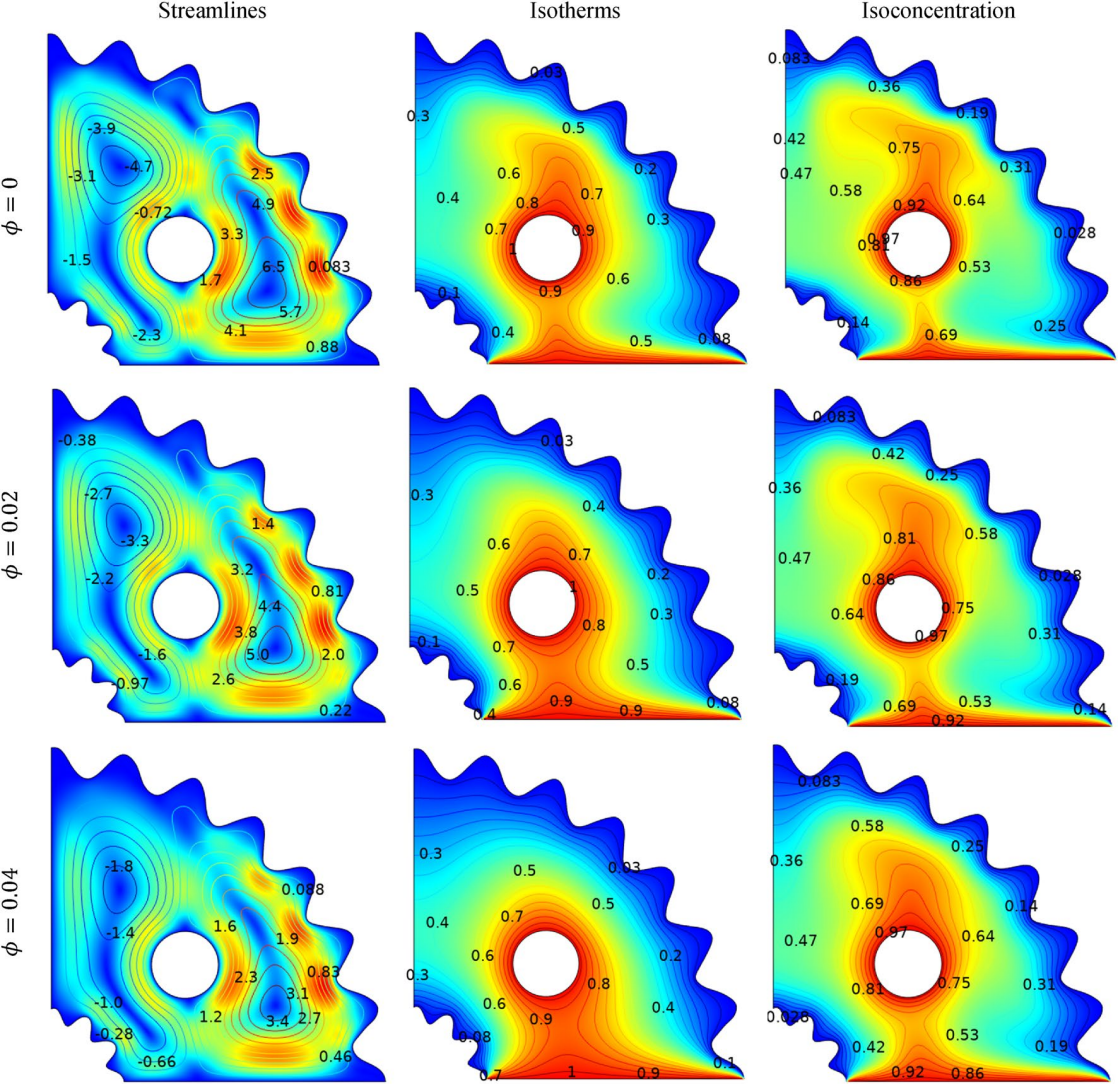
Impact of $\phi$ on streamlines, isotherms and isoconcentration.

In Fig. 9, the impact of the volume fraction ($\phi )$ of (Cu–Al_2_O_3_–H_2_O) hybrid nanofluid on streamlines, isotherms and isoconcentration is highlighted within the corrugated cavity, considering *Pr* = 6.2, *N* = *1*, *Ra* = ${10}^{5}$*, Le* = *2.5* and $Ha=25$. Regardless of $(\phi )$ values, the fluid motion exhibits both clockwise and counter-clockwise vortices. With an increase in the volume fraction of nanoparticles the fluid becomes more viscous leading to a reduction in buoyancy force and flow velocity. Consequently, stream function values decrease with the maximum strength of streamlines ${(\psi }_{max})$ dropping from 3.45 at $\phi =0$, to 1.25 at $\phi =0.04,$ same behavior is observed in ref.^44^. Negligible changes are occurred in isotherm and isoconcentration distribution for an increase in volume fraction of hybrid nanofluid as shown in Fig. 9 This is because, suspended nanoparticles increase the thermal conductivity of working fluids and hence the domination of conduction of working fluid and hence the domination of conduction mode heat transfers.

### Effects of Lewis number

**Figure 10:**
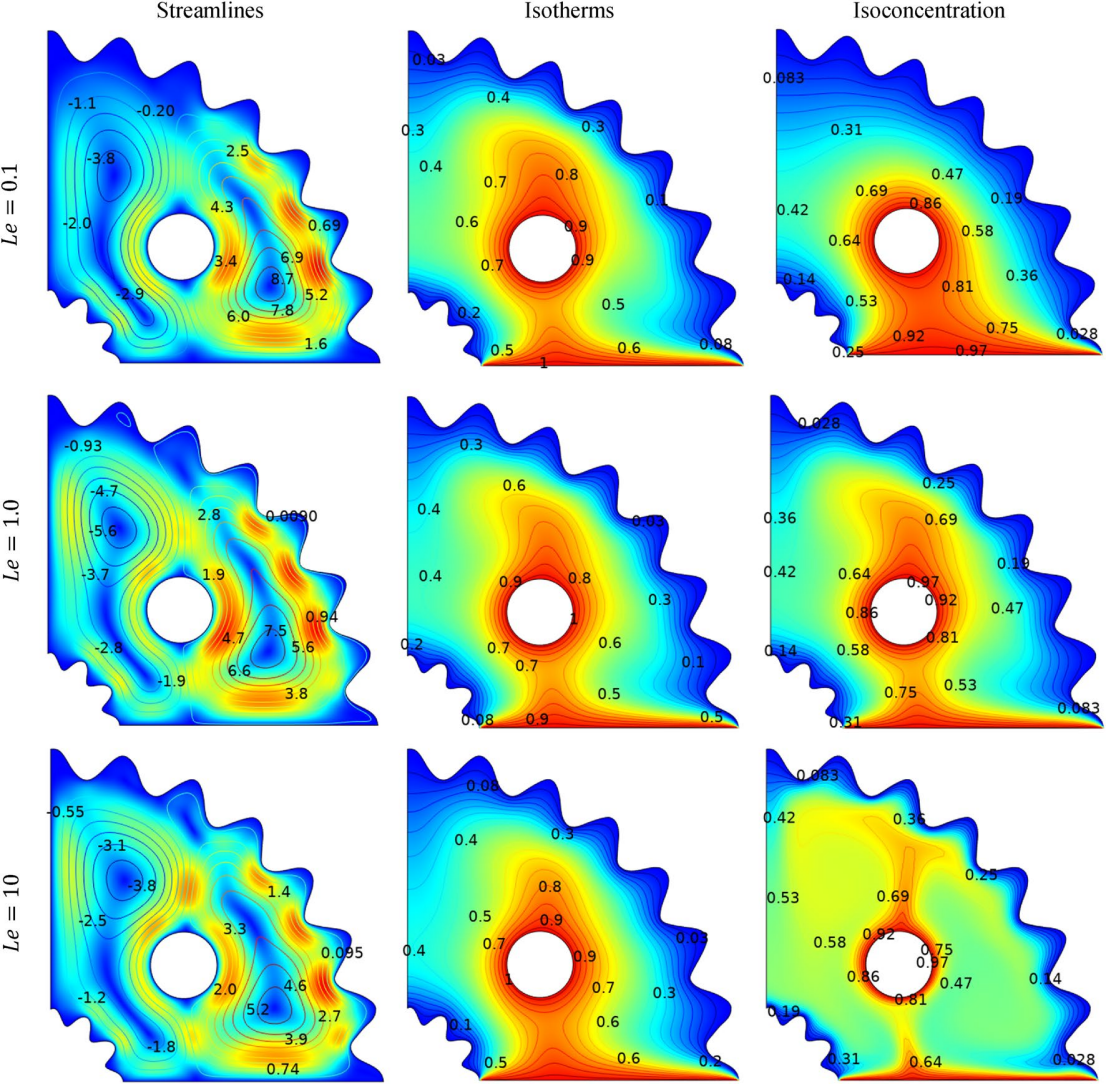
Impact of $Le$ on streamlines, isotherms and isoconcentration.

Figure 10 illustrates the impact of varying the Lewis number (*Le*) from 0.1 to 10 on momentum, temperature, and concentration distributions when Pr = 6.8, *Ha* = 25, ${Ra=10}^{4}$, *N* = 1 and ($\phi =0.04$). As, Lewis number (*Le*) governs the ratio of thermal diffusivity to mass diffusivity. Therefore, increased *Le* can be interpreted by the thermal diffusivity's dominance which restricts convective heat transport. It is also found that the maximum absolute stream function is ${\psi }_{max}=8.7$ at *Le* = *1* and decline to ${\psi }_{max}=5.2$ at *Le* = *10.* Increasing the Lewis number (Le) appears to have no effect on the isotherms. Figure 10 compares the positive trend in the magnitude of mass flux with respect to the Lewis number (Le). Lewis number define the ratio of thermal to mass diffusivity influences the thermal and mass diffusion characteristics. An increase in (Le) enhances thermal diffusivity but reduces mass diffusivity. As a result, an optimal zone is attained at Le = 0.1, leading to reduced mass dispersion. Meanwhile, a narrower region of isoconcentration is noticeable at Le = 10.

**Figure 11:**
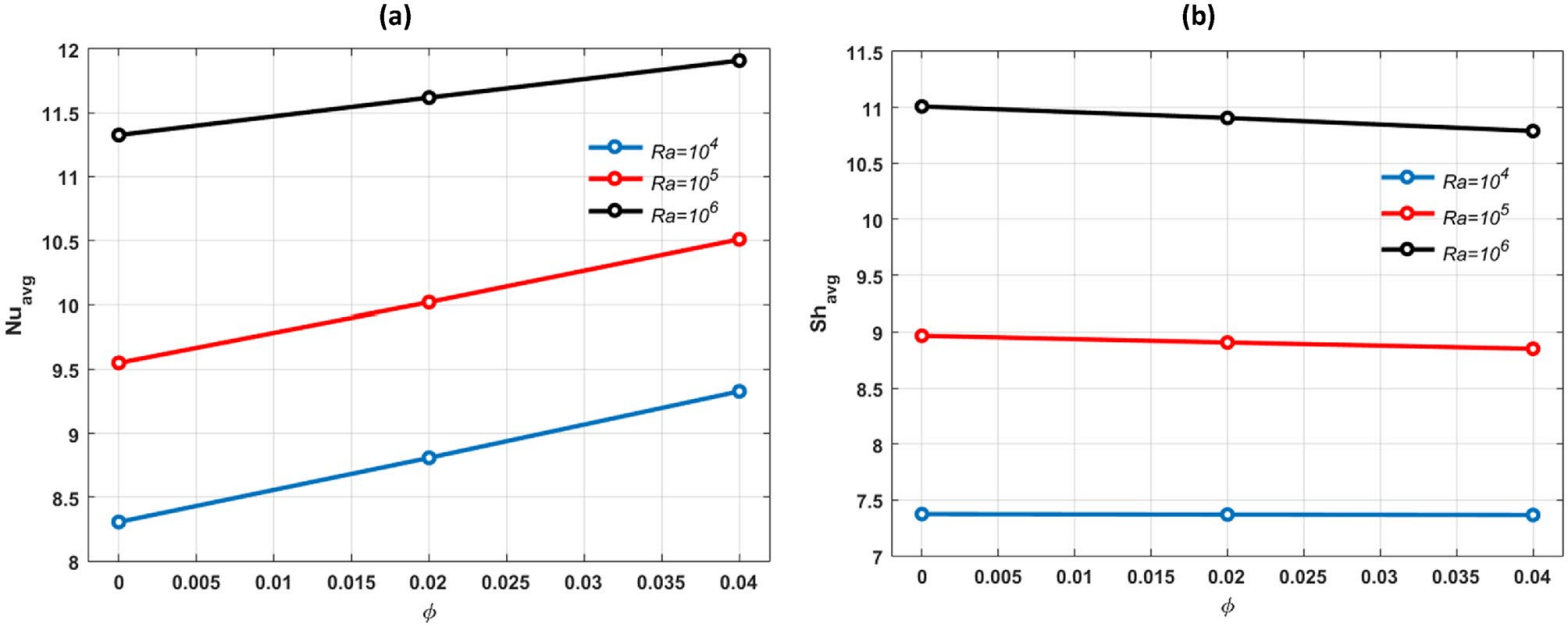
Effects of (**a**) $N{u}_{avg}$ and (**b**) $S{h}_{avg}$ for different values of $\varphi$ and $Ra.$

Figure 11a,b depict the acceleration of the average Nusselt number $(N{u}_{avg})$ left side and average Sherwood number $(S{h}_{avg}$) right side in relation to volume fraction of (Cu–Al_2_O_3_–H_2_O) hybrid nanofluid and for various Rayleigh number on the heated circular cylinder and on the heated bottom wall at fixed values of *Pr* = 6.2, *N* = *1*, $Ra={10}^{5}$ and $Ha=25.$ When *Ra* is high, free convection flows enhance and heat transfer rate rises. *Ra* raises the buoyancy force in the enclosure, and convection becomes the major mechanism of heat and mass transport raising $N{u}_{avg}$ and $S{h}_{avg}$.

**Figure 12:**
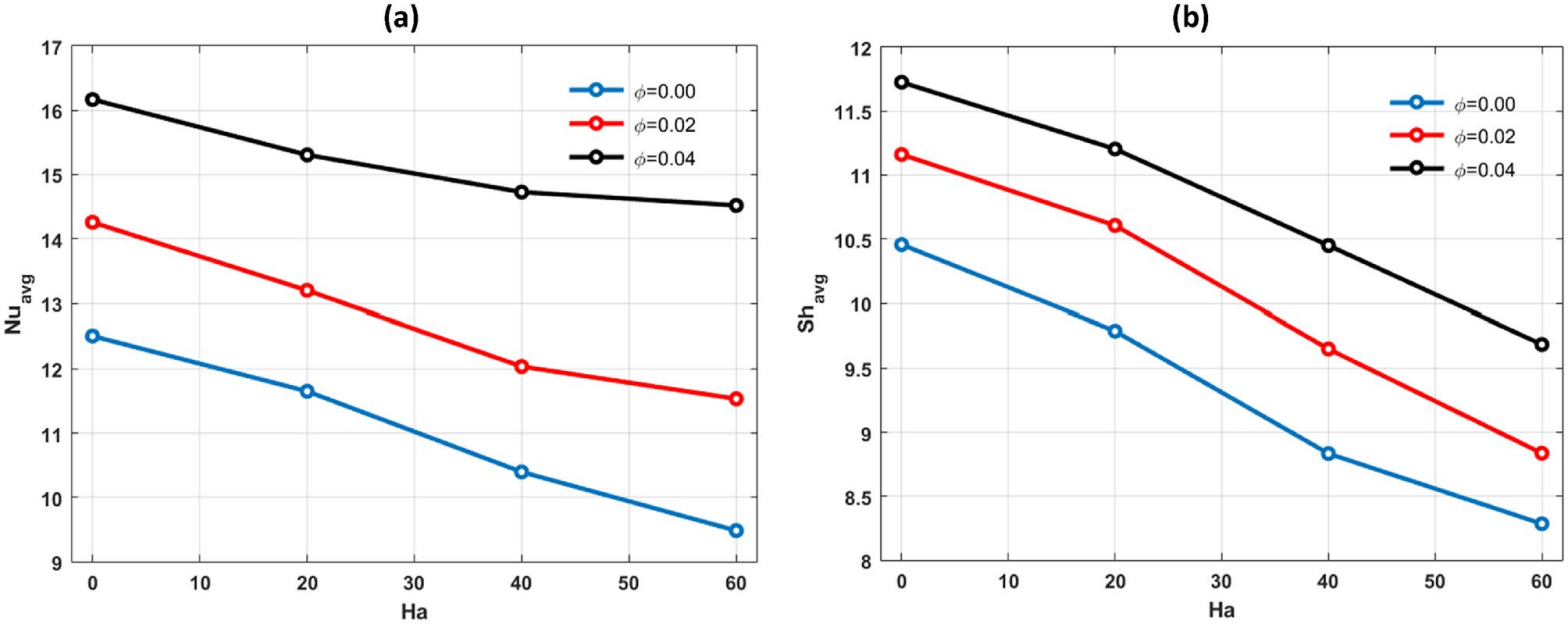
Effects of (**a**) $N{u}_{avg}$ and (**b**) $S{h}_{avg}$ for different values of $\varphi$ and $Ha.$

**Figure 13:**
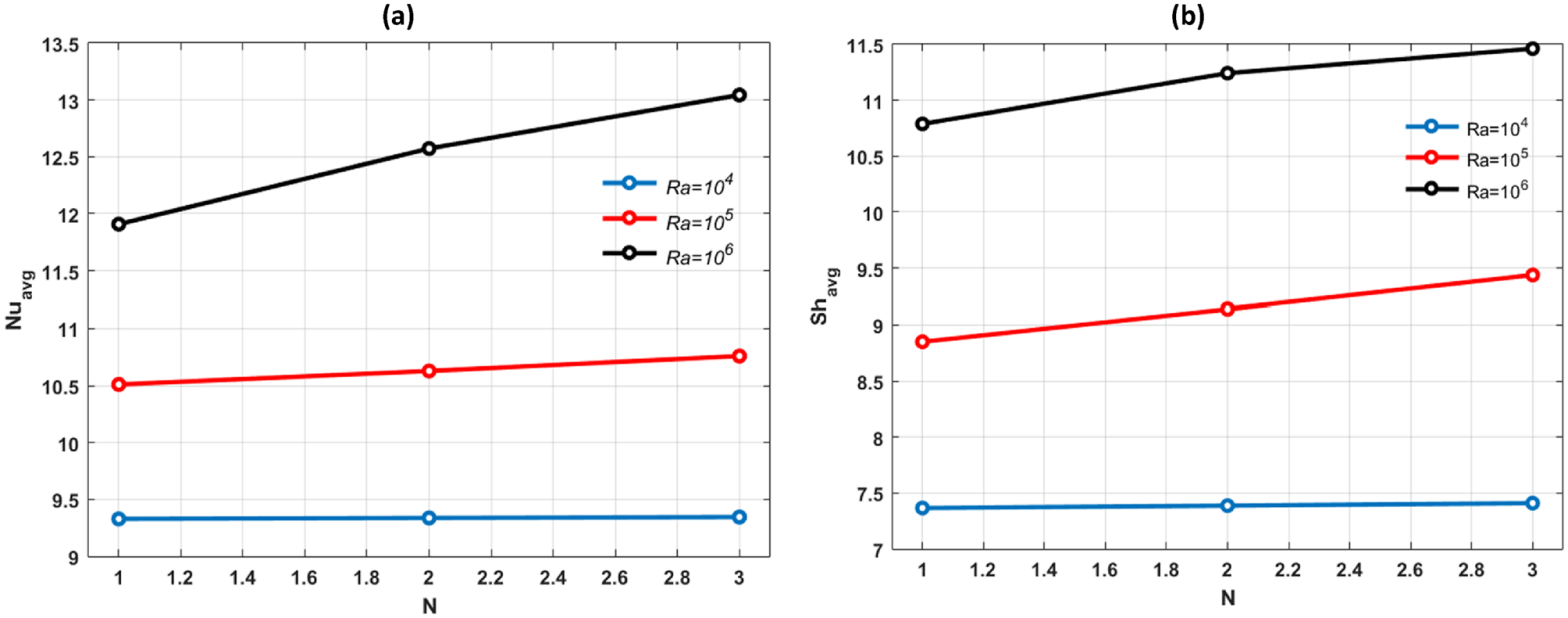
Effects of (**a**) $N{u}_{avg}$ and (**b**) $S{H}_{avg}$ for different values of $Ra$ and $N.$

Hartmann number impact on the average Nusselt number $(N{u}_{avg}$) and $(S{h}_{avg})$ is plotted in Fig. 12a,b. $(N{u}_{avg})$ and $(S{h}_{avg})$ decreases with supremacy of $Ha.$ With a strong magnetic field present the conduction mechanism gains strength leading to a slowdown in both heat and mass transfer rates. Figure 13a,b display the $(N{u}_{avg})$ and $(S{h}_{avg})$ in relation to the bouncy ratio (*N)* for different Rayleigh number including ${10}^{4}$, ${10}^{5}$ and ${10}^{6}$. The parameters in this figure are fixed to *Pr* = 6.2, *Ha* = 25, *n* = *11, Le* = *2.5* and $\phi =0.04.$ The findings show that bouncy ratio (*N*) has a positive effect on $(N{u}_{avg})$ and $(S{h}_{avg})$.

## Concluding remarks

The current study investigates novel physical phenomenon related to thermosolutal diffusion in water, specifically in the presence of (Cu–Al_2_O_3_) hybrid nanoparticles induced in a corrugated enclosure. The governing equations and boundary conditions are solved using the FEM to obtain a well-defined solution. Some salient outcomes of investigation are itemized as under

1. Convection mode grows as Rayleigh number rises but it diminishes as Lorentz forces rise.
2. The rate of heat and mass transmission as well as fluid motion all considerably increase as the Rayleigh number rises.
3. The components of fluid motion and transfer of heat are significantly reduced as the magnetic field influence increases. The Hartmann number also has an impact on the structure of the flow circulation.
4. It is discovered that the parameters under consideration have a significant impact on the fluid streamlines, isotherms and isoconcentrations behave inside the enclosure.
5. The heat transfer rate decreases while the mass transfer rate increases as the Lewis number rises.

The ongoing investigation can be expanded by examining the effects of porous media and entropy generation inside the enclosure. In addition, this work can also be extended by examining thermosolutal diffusion in water based ternary nano fluid instead of hybrid nano fluid flow.

## Data Availability

The datasets utilized and examined in the present study can be obtained from the corresponding author upon a reasonable request.

## References

[1] Sezai I, Mohamad AA (2000). Double diffusive convection in a cubic enclosure with opposing temperature and concentration gradients. Phys. Fluids.

[2] Gebhart B, Pera L (1971). The nature of vertical natural convection flows resulting from the combined buoyancy effects of thermal and mass diffusion. Int. J. Heat Mass Transf..

[3] Nishimura T, Wakamatsu M, Morega AM (1998). Oscillatory double-diffusive convection in a rectangular enclosure with combined horizontal temperature and concentration gradients. Int. J. Heat Mass Transf..

[4] Snoussi LB, Chouikh R, Guizani A (2005). Numerical study of the natural convection flow resulting from the combined buoyancy effects of thermal and mass diffusion in a cavity with differentially heated side walls. Desalination.

[5] Chouikh R, Snoussi LB, Guizani A (2007). Numerical study of the heat and mass transfer in inclined glazing cavity: Application to a solar distillation cell. Renew. Energy.

[6] Nikbakhti R, Rahimi AB (2012). Double-diffusive natural convection in a rectangular cavity with partially thermally active side walls. J. Taiwan Inst. Chem. Eng..

[7] Lee JW, Hyun JM (1990). Double-diffusive convection in a rectangle with opposing horizontal temperature and concentration gradients. Int. J. Heat Mass Transfer.

[8] Hyun JM, Lee JW (1990). Double-diffusive convection in a rectangle with cooperating horizontal gradients of temperature and concentration. Int. J. Heat Mass Transfer.

[9] Mahapatra TR, Pal D, Mondal S (2013). Effects of buoyancy ratio on double-diffusive natural convection in a lid-driven cavity. Int. J. Heat Mass Transfer.

[10] Costa VAF (2004). Double-diffusive natural convection in parallelogrammic enclosures filled with fluid-saturated porous media. Int. J. Heat Mass Transf..

[11] Ghorayeb K, Mojtabi A (1997). Double diffusive convection in a vertical rectangular cavity. Phys. Fluids.

[12] Mohamed A, El-Maghlany WM (2010). Numerical simulation of double-diffusive mixed convective flow in rectangular enclosure with insulated moving lid. Int. J. Therm. Sci..

[13] Abdallah MS, Zeghmati B (2014). Heat and mass transfer due to natural convection along a wavy vertical plate with opposing thermal and solutal buoyancy effects. Fluid Dyn. Mater. Process..

[14] Krishna MV, Jyothi K, Chamkha AJ (2018). Heat and mass transfer on unsteady, magnetohydrodynamic, oscillatory flow of second-grade fluid through a porous medium between two vertical plates, under the influence of fluctuating heat source/sink, and chemical reaction. Int. J. Fluid Mech. Res..

[15] Azad AK, Munshi MJH, Rahman MM (2013). Double diffusive mixed convection in a channel with a circular heater. Proc. Eng..

[16] Alamiri A, Khanafer K, Pop I (2009). Buoyancy-induced flow and heat transfer in a partially divided square enclosure. Int. J. Heat Mass Transf..

[17] Eshaghi S, Izadpanah F, Dogonchi AS, Chamkha AJ, Hamida MBB, Alhumade H (2021). The optimum double diffusive natural convection heat transfer in H-Shaped cavity with a baffle inside and a corrugated wall. Case Stud. Therm. Eng..

[18] Sourtiji E, Hosseinizadeh SF (2012). Heat transfer augmentation of magnetohydrodynamics natural convection in L-shaped cavities utilizing nanofluids. Therm. Sci..

[19] Manjunatha N, Sumithra R, Alessa N, Loganathan K, Selvamani C, Gyeltshen S (2023). Influence of temperature gradients and heat source in a combined layer on double component-magneto-marangoni-convection. J. Math..

[20] Nithyadevi N, Yang R-J (2009). Double diffusive natural convection in a partially heated enclosure with Soret and Dufour effects. Int. J. Heat Fluid Flow.

[21] Fatima N, Kousar N, Rehman KU, Shatanawi W (2023). Magneto-thermal convection in partially heated novel cavity with multiple heaters at bottom wall: A Numerical solution. Case Stud. Thermal Eng..

[22] Asghar Z, Ali N, Waqas M, Javed MA (2020). An implicit finite difference analysis of magnetic swimmers propelling through non-Newtonian liquid in a complex wavy channel. Comput. Math. Appl..

[23] Asghar Z, Ali N, Javid K, Waqas M, Khan WA (2021). Dynamical interaction effects on soft-bodied organisms in a multi-sinusoidal passage. Eur. Phys. J. Plus.

[24] Mansour MA, Ahmed SE, Rashad AM (2016). MHD natural convection in a square enclosure using nanofluid with the influence of thermal boundary conditions. J. Appl. Fluid Mech..

[25] Shamshuddin MD, Abderrahmane A, Koulali A, Eid MR, Shahzad F, Jamshed W (2021). Thermal and solutal performance of Cu/CuO nanoparticles on a non-linear radially stretching surface with heat source/sink and varying chemical reaction effects. Int. Commun. Heat Mass Transfer.

[26] Al-Kouz W, Medebber MA, Elkotb MA, Abderrahmane A, Aimad K, Al-Farhany K, Jamshed W (2021). Galerkin finite element analysis of Darcy–Brinkman–Forchheimer natural convective flow in conical annular enclosure with discrete heat sources. Energy Rep..

[27] Al-Kouz W, Aissa A, Koulali A, Jamshed W, Moria H, Nisar KS, Mourad A, Abdel-Aty AH, Motawi Khashan M, Yahia IS (2021). MHD darcy-forchheimer nanofluid flow and entropy optimization in an odd-shaped enclosure filled with a (MWCNT-Fe3O4/water) using galerkin finite element analysis. Sci. Rep..

[28] Rashad AM, Sivasankaran S, Mansour MA, Bhuvaneswari M (2017). Magneto-convection of nanofluids in a lid-driven trapezoidal cavity with internal heat generation and discrete heating. Numer. Heat Transfer Part A Appl..

[29] Rashad AM, Armaghani T, Chamkha AJ, Mansour MA (2018). Entropy generation and MHD natural convection of a nanofluid in an inclined square porous cavity: Effects of a heat sink and source size and location. Chin. J. Phys..

[30] Acharya N (2022). Magnetized hybrid nanofluid flow within a cube fitted with circular cylinder and its different thermal boundary conditions. J. Magn. Magn. Mater..

[31] Acharya N (2022). On the hydrothermal behavior and entropy analysis of buoyancy driven magnetohydrodynamic hybrid nanofluid flow within an octagonal enclosure fitted with fins: Application to thermal energy storage. J. Energy Stor..

[32] Acharya N, Chamkha AJ (2022). On the magnetohydrodynamic Al2O3-water nanofluid flow through parallel fins enclosed inside a partially heated hexagonal cavity. Int. Commun. Heat Mass Transfer.

[33] Cheng C-Y (2009). Combined heat and mass transfer in natural convection flow from a vertical wavy surface in a power-law fluid saturated porous medium with thermal and mass stratification. Int. Commun. Heat Mass Transfer.

[34] Acharya N (2022). Effects of different thermal modes of obstacles on the natural convective Al2O3-water nanofluidic transport inside a triangular cavity. Proc. Inst. Mech. Eng. C J. Mech. Eng. Sci..

[35] Acharya N (2021). On the flow patterns and thermal control of radiative natural convective hybrid nanofluid flow inside a square enclosure having various shaped multiple heated obstacles. Eur. Phys. J. Plus.

[36] Takabi B, Salehi S (2014). Augmentation of the heat transfer performance of a sinusoidal corrugated enclosure by employing hybrid nanofluid. Adv. Mech. Eng..

[37] Parveen R, Mondal P, Mahapatra TR (2021). "Double diffusive magnetohydrodynamic (MHD) natural convection and entropy generation in a discretely heated inclined dome-shaped enclosure filled with cu-water nanofluid. J. Nanofluids.

[38] Multiphysics C (1998). Introduction to COMSOL multiphysics extregistered. COMSOL Multiphys..

[39] Shafqat H, Shoeibi S, Armaghani T (2021). Impact of magnetic field and entropy generation of Casson fluid on double diffusive natural convection in staggered cavity. Int. Commun. Heat Mass Transfer.

[40] Lizardi A, Terres H, López R, Vaca M, Chávez S, Lara A, Morales JR (2017). Experimental and numerical analysis of convective flow in a square cavity with internal protuberances. J. Phys. Conf. Ser..

[41] Koulali A, Abderrahmane A, Jamshed W, Hussain SM, Sooppy Nisar K, Abdel-Aty A-H, Yahia IS, Eid MR (2021). Comparative study on effects of thermal gradient direction on heat exchange between a pure fluid and a nanofluid: Employing finite volume method. Coatings.

[42] Koulali A, Sahi A, Meziani B, Aissa A, Sadaoui D, Ali HM (2023). CFD analysis of natural convection between two superposed fluids: Role of corrugated bottoms. Chem. Eng. Commun..

[43] Nayak MK, Karimi N, Chamkha AJ, Dogonchi AS, El-Sapa S, Galal AM (2022). Efficacy of diverse structures of wavy baffles on heat transfer amplification of double-diffusive natural convection inside a C-shaped enclosure filled with hybrid nanofluid. Sustain. Energy Technol. Assess..

[44] Parveen R, Mahapatra TR (2019). Numerical simulation of MHD double diffusive natural convection and entropy generation in a wavy enclosure filled with nanofluid with discrete heating. Heliyon.

